# Cutaneous pigmentation modulates skin sensitivity via tyrosinase-dependent dopaminergic signalling

**DOI:** 10.1038/s41598-017-09682-4

**Published:** 2017-08-23

**Authors:** Kentaro Ono, Chi T. Viet, Yi Ye, Dongmin Dang, Suzuro Hitomi, Takashi Toyono, Kiyotoshi Inenaga, John C. Dolan, Brian L. Schmidt

**Affiliations:** 1 Bluestone Centre for Clinical Research, New York University College of Dentistry, New York, NY 10010 USA; 2 Department of Oral Maxillofacial Surgery, New York University College of Dentistry, New York, NY 10010 USA; 3 Division of Physiology, Kyushu Dental University, Kitakyushu, Fukuoka, 803-8580 Japan; 4 Division of Oral Anatomy, Kyushu Dental University, Kitakyushu, Fukuoka, 803-8580 Japan

## Abstract

We propose a new mechanism of sensory modulation through cutaneous dopaminergic signalling. We hypothesize that dopaminergic signalling contributes to differential cutaneous sensitivity in darker versus lighter pigmented humans and mouse strains. We show that thermal and mechanical cutaneous sensitivity is pigmentation dependent. Meta-analyses in humans and mice, along with our own mouse behavioural studies, reveal higher thermal sensitivity in pigmented skin relative to less-pigmented or albino skin. We show that dopamine from melanocytes activates the D_1_-like dopamine receptor on primary sensory neurons. Dopaminergic activation increases expression of the heat-sensitive TRPV1 ion channel and reduces expression of the mechanically-sensitive Piezo2 channel; thermal threshold is lower and mechanical threshold is higher in pigmented skin.

## Introduction

Pigmentation in human skin can affect thermoregulation and photoprotection from ultraviolet (UV) radiation^1^. Dark skin reduces UV damage to the DNA, however, for some non-equatorial populations there may be selective pressure for lighter pigmented skin to facilitate cutaneous photosynthesis of vitamin D_3_ under low UVB radiation intensity^1^. Cutaneous pain sensitivity is affected by selective forces different than the selective forces that affect pigmentation^2, 3^. Human studies have reported inconsistent racial/ethnic differences in thermal and ischemic pain^4–14^. Racial/ethnic pain differences are reportedly associated with hormonal regulation in the HPA-axis^8, 10^ and single nucleotide polymorphisms (SNPs) in genes of μ-opioid receptor (*OPRM1*)^13^.

Human skin shade is altered by size and density of melanin particles, trauma, UV exposure and melanocyte-stimulating hormone (MSH)^15, 16^. Racial differences in melanin synthesis are primarily attributed to the amount and catalytic activity of the copper-containing enzyme tyrosinase^17^, which catalyzes critical steps of mammalian melanogenesis^15, 16^. Interestingly, the locus of the mouse tyrosinase gene *Tyr* (chromosome 7, 49 cM) is adjacent to the detected location of a gene that affects heat sensitivity^18^. Animal studies demonstrate that functional knockouts of genes for the α-MSH receptor (MC1R) and the tyrosinase-related protein 1 (TYRP1) produce lower basal heat sensitivity; these two genes are predominantly expressed in melanocytes^19–21^. Skin melanocytes form tight contacts with cutaneous nerves^22^ and secrete the catecholamine precursor L-dopa^23^, which is also synthesized during melanogenesis by tyrosinase^15, 16^. These studies suggest that skin melanocytes peripherally regulate sensation.

Our meta-analysis in humans and mice reveals greater heat sensitivity in mice and in humans with darker skin. We confirmed these findings in mice with behavioural tests. The heat sensitivity mechanism in humans and in mice involves pigmentation-dependent dopaminergic signalling from skin melanocytes which modulate gene expressions of the heat-sensitive TRPV1 channel and the mechano-sensitive Piezo2 channel in primary sensory neurons via D_1_-like receptors.

## Results

### Cutaneous heat sensitivity is higher in dark skinned people compared with light skinned people

**Figure 1:**
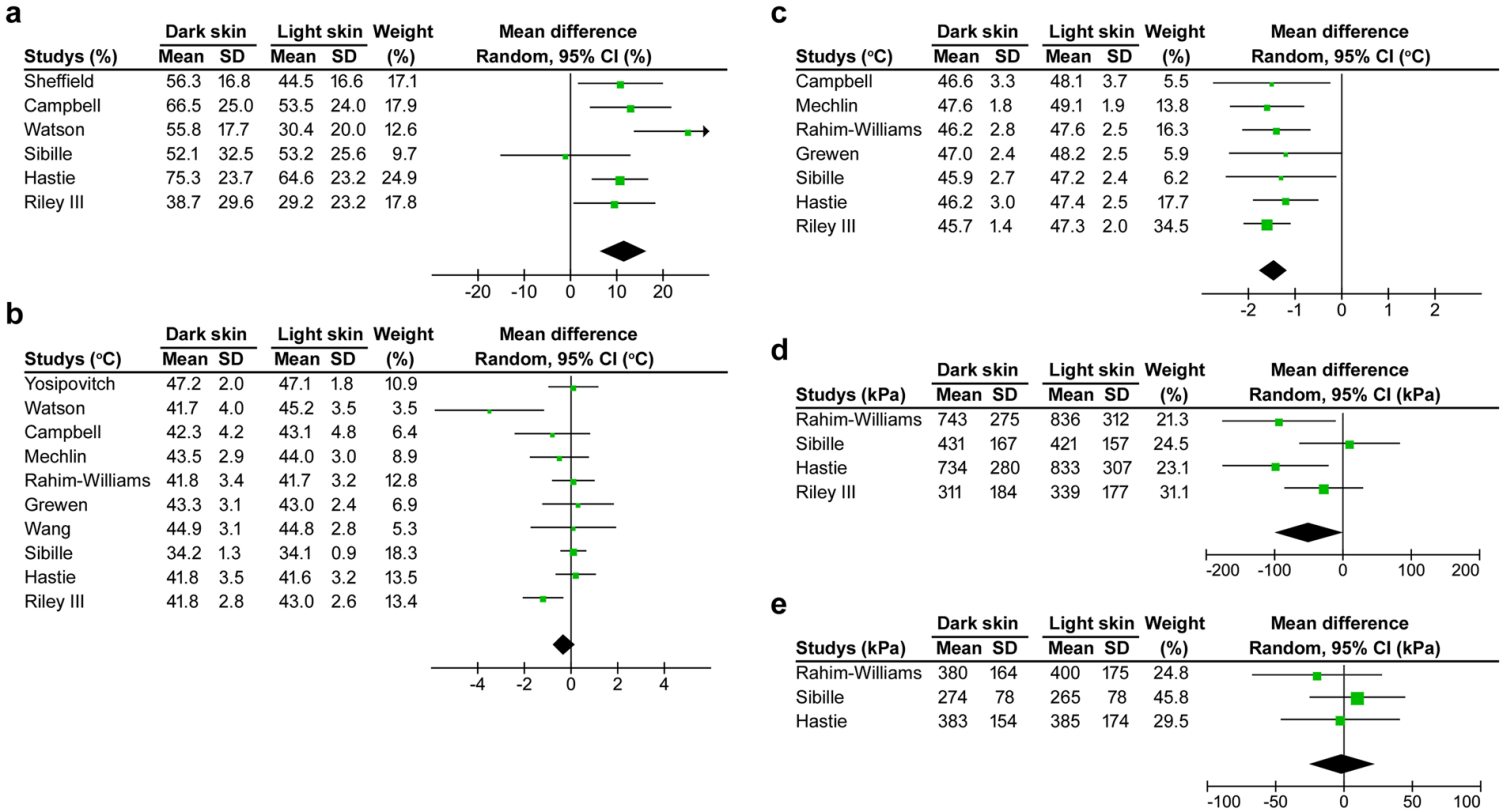
Forest plots of the mean differences in heat and pressure pain sensitivities between dark and light skin human cohorts. Study name is defined as the last name of the first author and all studies are listed in Table S1. (**a**) Heat pain intensity (% of visual analogue scale) in the forearm to 48 or 49 °C stimulation is higher in dark skin cohort than light skin cohort (6 studies). Heterogeneity among the studies was moderate (*I* ^2^ = 44, *P* < 0.11). (**b**) There is no difference in heat pain threshold (the first painful temperature) in the forearm between the two cohorts (10 studies). However, there is a trend toward lower heat pain thresholds in the dark skin cohort. Heterogeneity among the studies was moderate (*I* ^2^ = 46, *P* < 0.06). (**c**) Heat pain tolerance (intolerance temperature) in the forearm is lower in dark skin cohort than light skin cohort (7 studies). There is no heterogeneity among the studies (*I* ^2^ = 40, *P* < 0.98). (**d**) There is no difference in the pain pressure threshold in trapezius muscle (4 studies). However, there is a trend toward lower pressure threshold of the dark skin cohort than that of the light skin cohort (*P* = 0.05). Heterogeneity among the studies is moderate (*I* ^2^ = 46, *P* < 0.13). (**e**) There was no difference in the pain pressure threshold in the masseter muscle (3 studies). There is no heterogeneity among the studies (*I* ^2^ = 40, *P* < 0.62).

To reveal any correlation between skin shade and cutaneous sensitivity in humans, we performed a meta-analysis of racial/ethnic differences in cutaneous heat and mechanical sensitivities in healthy human subjects. Our initial Pubmed search revealed 80 unique abstracts with appropriate keywords (see Methods). Twenty-five of these abstracts were not pain-related and were excluded. The remaining 55 full-text articles in English were collected and reviewed to determine eligibility. A total of 37 full-text articles were excluded for the following reasons: the study did not compare racial/ethnic difference in pain sensitivities in humans (22 articles), the study did not include a healthy control group (6 articles), the study did not show mean values of heat or mechanical pain sensitivity (7 articles), and the study sample was composed solely of children (2 articles). Study evaluation, extraction and validation were conducted independently by two co-authors (S.H. and K.I.). Data from the remaining 18 articles were extracted by the first author (K.O.). Racial/ethnic groups were divided into light and dark skin cohorts (von Luschan skin tone: 1–17 and 18–36, respectively) based on the map of native skin colour distributions^24^. During our coding process 6 additional articles were excluded: one article because of the mixture of light and dark skin cohorts, three articles because they contained only one cohort, one article because it did not contain a description of standard deviation and one article in which a heat pain test was performed on the palm skin (only studies on forearm skin were included). Because two articles used the same database, one of these articles was excluded from the meta-analysis. Eleven articles^4–14^ were utilized to generate the meta-analysis (Supplementary Table S1). All studies report heat pain intensity (visual analogue scale) to a heat stimulation of 48 or 49 °C and/or heat pain threshold (the first painful temperature) and heat pain tolerance (the first intolerance temperature) to a ramp heat stimulation in the forearm skin. In the heat pain intensity analysis, the dark skin cohort’s mean was higher than the light skin cohort’s value (Cochran *Q*, *P* < 0.00001; Fig. 1a). In the heat pain threshold analysis, there was no significant difference in mean differences between the light and dark skin shade cohorts (*P* = 0.24) although a trend toward a lower heat pain threshold in the dark skin cohort is evident (Fig. 1b). In the heat pain tolerance analysis, the dark skin cohort mean was lower than the light skin cohort value (*P* < 0.00001; Fig. 1c). Of the 11 studies in our analysis, 4 evaluate pressure pain threshold (the first onset of pain from the pressure stimulus) in the trapezius and/or masseter muscle. No significant differences were identified in pressure pain thresholds in either region between the two cohorts (*P* = 0.05 and 0.92, respectively; Fig. 1d,e). Our human meta-analyses related to heat sensitivity suggest that the dark skin cohort show increased heat sensitivity relative to the light skin cohort. Pain sensitivity resulting from a pressure stimulus is not significantly different between the dark and light skin cohorts.

### Pigmented mice show higher sensitivity (lower threshold to respond) to thermal and capsaicin stimulus compared to unpigmented mice

**Figure 2:**
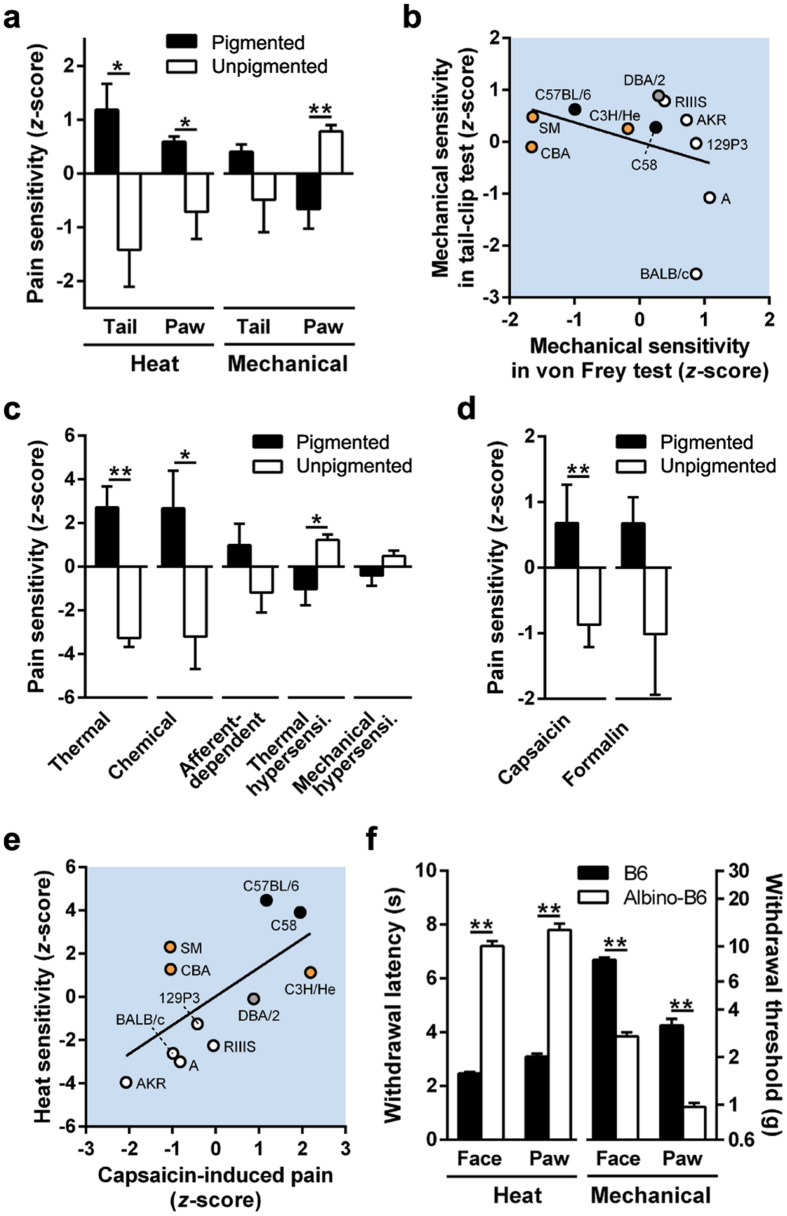
Pigmented and unpigmented mice display differences in cutaneous sensitivities. All bars show mean ± s.e.m. The colour of each plot represents the body colour of the inbred mouse strain. (**a**) Heat and mechanical sensitivities in the tail and paw of pigmented and unpigmented male mice (6 and 5 strains, respectively). Increased pain sensitivity to stimuli is depicted by positive *z*-scores. (**b**) There is no correlation between mechanical sensitivities as measured by tail-clip and von Frey tests (*r* = −0.38, *P* < 0.25). (**c**) The sensitivities across 5 different pain modalities (thermal, chemical, afferent-dependent, thermal hypersensitivity and mechanical hypersensitivity) in pigmented and unpigmented mouse groups (6 and 5 strains, respectively) are displayed. The latter three modalities are pathological pain sensitivities in neuropathic and/or inflammatory models. *P < 0.05 and **P < 0.01, in *t*-test. (**d**) Capsaicin and formalin sensitivities between pigment and unpigmented mouse groups (6 and 5 strains, respectively). **P < 0.01, in *t*-test. (**e**) There is a positive correlation between capsaicin-induced pain and heat sensitivity. Pearson *r* = 0.81, *P* < 0.01. Pigmented mouse strains show higher thermal and capsaicin sensitivities. (**f**) Withdrawal latency to radiant heat stimulation and threshold to von Frey filaments in the whisker pad (face) and paw of B6 (*n* = 5 and 10, respectively) and albino-B6 (*n* = 65 and 30, respectively) female mice. *P < 0.05 and **P < 0.01, in *t*-test.

We propose that experimental cutaneous pain thresholds (thermal and mechanical) in mouse strains possessing different amounts of cutaneous pigmentation can provide insight into the mechanisms underlying pigmentation-related pain modulation in humans. To investigate pain threshold and pigmentation in mice we used an online database that included 21 nociceptive assays in 11 inbred mouse strains^25^. Strains were divided into two body-colour groups as follows: pigmented group including C3H/He, C57BL/6, C58, CBA, DBA/2 and SM, and an unpigmented group including 129P3, A, AKR, BALB/c and RIIIS. In the pigmented and unpigmented mouse groups, pigment phenotype correlates with the genomic segment containing the *Tyr* gene^26, 27^. Our analysis of the publicly available database demonstrated significantly higher thermal sensitivity and lower von Frey mechanical sensitivity in the pigmented group relative to the unpigmented group; no differences were found between the groups in tail-clip sensitivity (Fig. 2a). No correlation was found between von Frey and tail-clip mechanical sensitivity (Fig. 2b); we regard the assays (and respective stimuli) utilized to obtain these data as different stimulus modalities. Von Frey filaments exert a light pricking pressure while the tail-clip induces a strong shearing pressure. Multivariate analysis of 21 nociceptive and hypersensitivity assays among inbred mouse strains reveals five different pain modalities: thermal, chemical, afferent dependent, thermal hypersensitivity and mechanical hypersensitivity^28^. Among the five modalities, the pigmented group show higher thermal and chemical sensitivities than the unpigmented group; however, for the latter three pain modalities, the pigmented group demonstrate lower thermal and chemical sensitivity compared to the unpigmented group (Fig. 2c). Interestingly, capsaicin sensitivity, a form of chemical-induced nociception, was higher in the pigmented group than the unpigmented group while formalin sensitivity did not show statistical significance (Fig. 2d). Capsaicin sensitivity was positively correlated with heat sensitivity among the inbred mouse strains (Fig. 2e), consistent with a previous study showing a positive correlation between TRPV1 levels and cutaneous heat sensitivity across different mouse strains^29^.

To minimize differences in genetic background among mouse strains, we compared cutaneous sensitivities between C57BL/6 (B6) and B6(Cg)-*Tyr* ^*c*−*2J*^ (albino-B6); the latter lacks functional tyrosinase activity due to a mutation in the tyrosinase gene, *Tyr* ^23^. In the whisker pad (face) and hind paw, wild type pigment B6 exhibited shorter withdrawal latency to heat stimulation but higher withdrawal threshold with von Frey-mechanical stimulation relative to albino-B6 (Fig. 2 f). From our analysis of the online database and our experimental behavioural findings we infer that pigmentation alters cutaneous sensitivity (heat, chemical and mechanical), independent of skin type (hairy or glabrous) and segment (trigeminal and sciatic nerve innervations), in humans and mice.

### Dopamine signalling from melanocytes modulates cutaneous thermal and mechanical sensitivities via D_1_-like receptor

**Figure 3:**
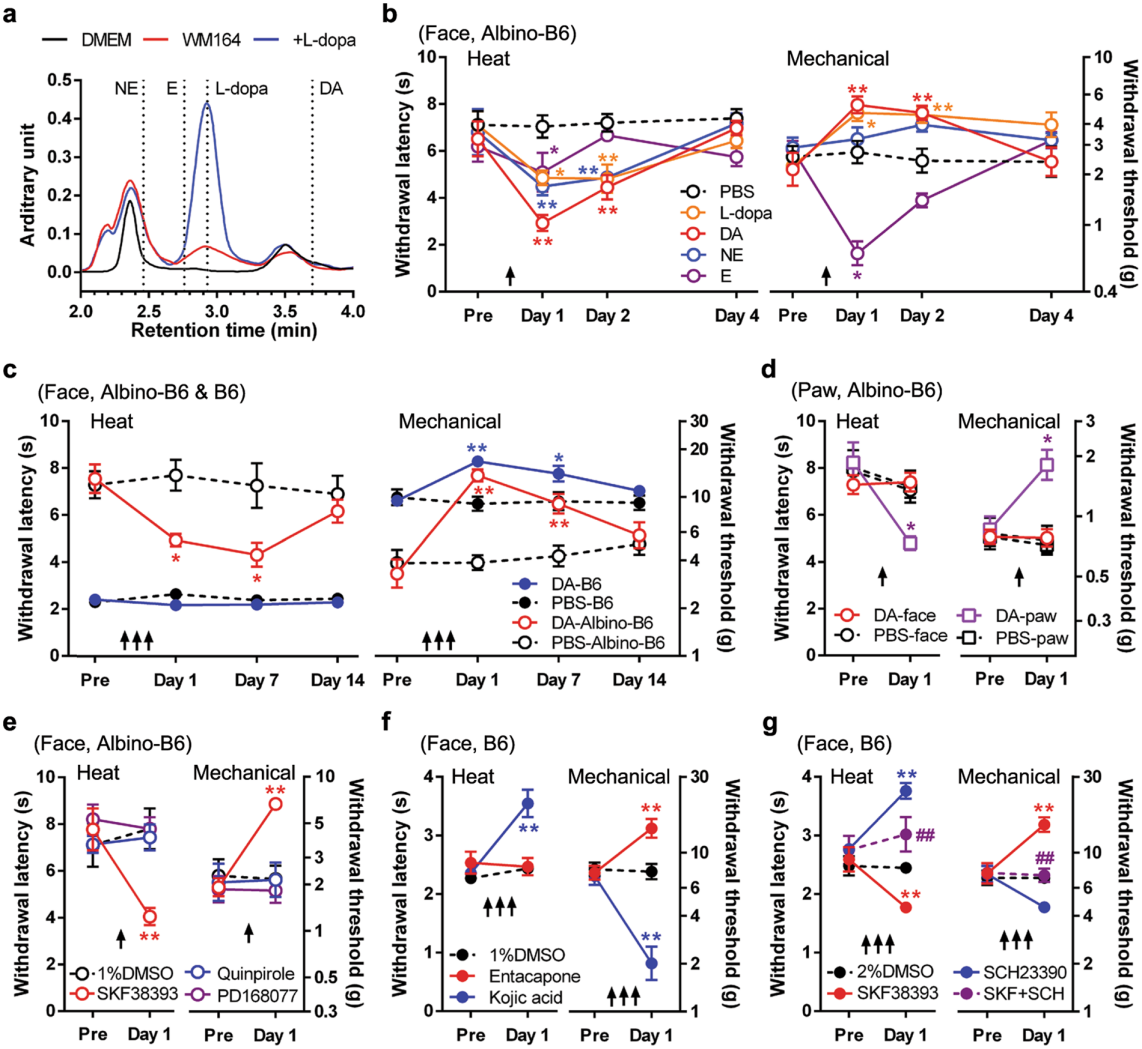
Pigmentation-dependent cutaneous sensations are mediated by skin dopaminergic signalling. All plots show mean ± s.e.m. Each animal experimental group, n = 5. Arrows indicate time of drug injection. (**a**) L-dopa secretion from melanoma cells. High-performance liquid chromatography of supernatant from human melanoma WM164 cells (red line). DMEM is the culture medium (black line). Dashed lines show peaks of norepinephrine (NE), epinephrine (E), L-dopa (+L-dopa, blue line, at 500 μM) and dopamine (DA). The experiment was replicated 5 times. (**b**) Withdrawal latency to radiant heat stimulation (heat) and threshold by von Frey-mechanical stimulation (mechanical) following injection of L-dopa, DA, NE, E (each 50 nmol in phosphate-buffered saline (PBS)) into the face of albino-B6 mice. *P < 0.05, **P < 0.01, *vs* PBS, in Sidak’s *post hoc* test following two-way ANOVA test. (**c**) Effects of repetitive DA injection (3 times) on withdrawal latency to heat stimulation and mechanical withdrawal threshold in the face of B6 and albino-B6. In B6, a single dopamine injection did not show any effect on cutaneous sensations one day following the injection (data not shown). Repeated injections of dopamine had no effect on heat sensitivity on days 1 and 7 although it reduced mechanical sensitivity. *P < 0.05, **P < 0.01, *vs* PBS, in Sidak’s *post hoc* test, following two-way ANOVA test. (**d**) Effects of DA injection into the face or hind paw on cutaneous sensitivities in the hind paw of albino-B6. (**e**) Effects of the D_1/5_ agonist SKF38393, the D_2/3_ agonist quinpirole, and the D_4_ agonist PD168077 (15 nmol each) in the face of albino-B6. (**f**) Effects of entacapone (50 nmol, an inhibitor of dopamine degradation) and kojic acid (1.5 μmol, an inhibitor of tyrosinase) in B6. (**g**) Effects of SKF38393 (15 nmol) and/or the D_1/5_ antagonist SCH23390 (50 nmol) in B6. *P < 0.05, **P < 0.01, *vs* each control (PBS or 1–2% dimethyl sulfoxide (DMSO)) and ^##^ *P* < 0.01, *vs* SKF38393, in two-way ANOVA test.

We showed L-dopa release (68 ± 6 μM, 5 independent experiments) from human melanoma cells, without catecholamines (Fig. 3a). We demonstrated an incremental increase (104 ± 14 μM, *P* < 0.05, *t-*test, 9 independent experiments) in L-dopa following enhancement of tyrosinase activity by applying α-MSH.

To examine whether L-dopa alters cutaneous sensitivities, we injected L-dopa into the facial subcutaneous tissue of albino-B6 mice that lack peripheral L-dopa secretion^23^. Compared with control injection, L-dopa reduced heat latency and increased mechanical threshold on days 1–2 following injection (Fig. 3b), demonstrating a pattern which follows pigmentation status (Fig. 2 f). Importantly, the subcutaneous L-dopa injection did not show any changes in heat or mechanical sensitivities for 2 hours after the injection, which differs from the acute effects of catecholamines on heat sensitivity (Supplementary Fig. S1a). Similarly, in healthy human subjects, L-dopa administration does not alter acute pain threshold in the cold-pressure test^30^. Accordingly, the changes in cutaneous sensitivities on the day following the L-dopa injection are presumed to be induced by bioactive catecholamines - enzymes required for conversion of L-dopa to catecholamines are present in the skin^31^. Importantly, dopamine showed the strongest L-dopa-like effects (reduction of heat withdrawal latency and increment of mechanical withdrawal threshold) on days 1–2 relative to the other catecholamines (Fig. 3b). We infer from these findings that dopamine is the primary agent driving changes in sensitivity. Cutaneous sensitivity changes were prolonged by repetitive dopamine injection (Fig. 3c), compared with a single injection (Fig. 3b). Dopamine injected into the paw reduced the heat withdrawal latency and increased mechanical withdrawal threshold (Fig. 3d). Injection of dopamine into the face did not change sensitivity in the paw (Fig. 3d); apparently the exogenous dopaminergic sensory modulation is locally mediated in albino-B6, without systemic and central effects. To identify which dopamine receptor subtypes are activated during dopaminergic sensory modulation, we subcutaneously injected subtype-specific dopamine receptor agonists into the face. The D_1_-like (D_1_/D_5_) receptor agonist SKF38393 reduced heat withdrawal latency and increased mechanical withdrawal threshold on day 1. D_2_-like receptor agonists quinpirole and PD168077 did not change (Fig. 3e), indicating involvement of D_1_-like receptor in the doperminargic sensory modulation.

Pigment B6 mice did not show any changes in heat or mechanical sensitivity on day 1 after a single injection of dopamine in the face (data not shown) although the same procedure in albino-B6 showed significant changes (Fig. 3b). Subsequently, we performed two additional drug injections (a total of three injections) in pigment B6 mice to determine whether repeated dopamine injection could modulate mechanical or heat sensitivity. Through repeated dopamine injection, mechanical withdrawal threshold was significantly increased on days 1 and 7 while heat withdrawal latency was unchanged in pigmented B6 mice (Fig. 3c). We infer from these results that exogenous dopamine was less effective in modulating facial sensitivities in pigment B6 mice than in albino-B6 mice; wild-type pigment B6 mice exhibit constitutive L-dopa secretion into the skin^23^. To enhance endogenous dopamine in pigment B6, Entacapone, an inhibitor of dopamine degradation, was injected into the face. The drug increased the mechanical withdrawal threshold, but did not change the heat withdrawal latency in pigment B6 mice (Fig. 3f). Repetitive injections of SKF38393 reduced heat latency and increased mechanical threshold in B6 (Fig. 3g), similar to findings in albino-B6. Next, to inhibit endogenous dopamine signalling in pigment B6, inhibitors of tyrosinase (kojic acid) and a highly selective D_1_-like receptor antagonist (SCH23390) were injected separately into the face. Both drugs prolonged heat withdrawal latency and decreased mechanical withdrawal threshold (Fig. 2f,g); the mechanical withdrawal threshold in the treated pigment B6 group approached the threshold of the albino-B6 group (Fig. 2f). We conclude that endogenous dopamine modulates cutaneous sensitivity via D_1_-like receptor activation.

Applications of dopamine at 10 μM as well as SKF38393, quinpirole and PD168077 at 30 μM induced a Ca^2+^ response in cultured trigeminal ganglion (TG) neurons of pigment B6 and albino-B6 mice (Supplementary Fig. S2a). The percentage of neurons that responded to either SKF3839 or dopamine was similar in both B6 and albino-B6 mice; neuronal response was measured with Ca^2+^ imaging (Supplementary Fig. S2b); SKF38393 and dopamine did not induce significant differences in the amplitudes of the Ca^2+^ response (Supplementary Fig. S2c). On the other hand, quinpirole- or PD168077-induced Ca^2+^ responses in fewer neurons (10–25% of total cells) and the response was smaller relative to dopamine-induced Ca^2+^ responses (Supplementary Fig. S2b,c). We infer from these results that dopamine-induced Ca^2+^ response is predominantly mediated by D_1_-like receptors. SKF38393-induces a Ca^2+^ response in cultured B6 dorsal root ganglion neurons that are mediated by Ca^2+^ influx from the extracellular space via TRPV1, as opposed to intracellular Ca^2+^ stores^32^. The excitation effect of SKF38393 on cultured sensory neurons is consistent with the SKF38393-mediated heat hypersensitivity in the acute phase (Supplementary Fig. S2b). Together with D_1_ and D_5_ immunoreactivities in TG neurons (Supplementary Fig. S2d,e), the acute behavioural and cellular responses by dopamine receptor subtype specific agonists suggest that almost all subtypes are expressed in peripheral sensory nerves of mice, consistent with many early studies in TG and dorsal root ganglion neurons^32–37^. Pigment B6 sensory neurons demonstrated lower expression of D_1_, (but not D_5_) compared with albino-B6 neurons, as measured by immunofluorescence (Supplementary Fig. S2d,e); this finding is consistent with reduced responsiveness of pigment B6 mice to SKF38393, as measured by Ca^2+^ imaging (Supplementary Fig. S2b). The lower expression of D_1_ in sensory neurons of pigment B6 might explain the reduced effect of dopaminergic stimulation on sensory modulation.

### Dopamine signalling modulates *Trpv1* and *Piezo2* expressions in sensory neurons

**Figure 4:**
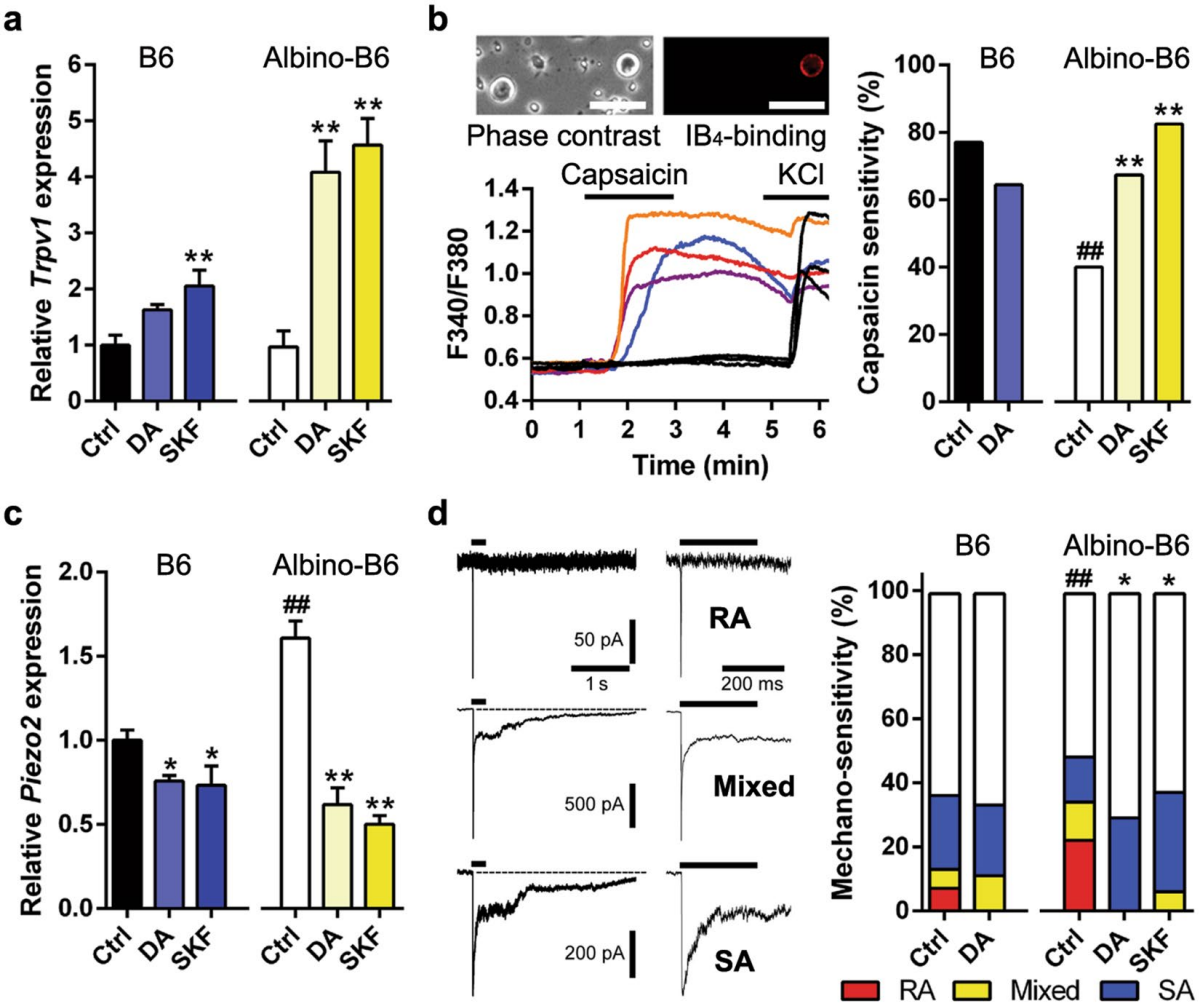
Dopamine modulates pain-related gene expression in sensory neurons. (**a**) *Trpv1* levels in the trigeminal ganglion (TG) following repeated injection of PBS (control (Ctrl), n = 4), dopamine (DA, n = 6) or SKF39393 (SKF, n = 4). *P < 0.05, **P < 0.01, *vs* control, in *t*-test. All bars show mean ± s.e.m. (**b**) Capsaicin sensitivity in IB_4_-positive TG neurons following Ctrl, DA (10 μM) or SKF (30 μM) incubation. Photomicrographs are representative phase contrast and IB_4_-fluorescence images. Scale bar, 50 μm. Traces are representative Ca^2+^ responses in TG neurons. Ctrl and DA in B6: n = 27/35 and 29/45, respectively. Ctrl, DA and SKF in albino-B6: n = 12/30, 37/55 and 33/40, respectively. **P < 0.01, *vs* Ctrl, ^##^ *P* < 0.01, *vs* B6, in Fischer’s test. (**c**) *Piezo2* levels in the TG following repeated injection of PBS, DA or SKF. Sample preparation and analysis were the same as **A**. ^##^ *P* < 0.01, *vs* B6. (**d**) Mechanically-activated currents (rapidly-adapted (RA), slowly-adapted (SA) and mixed types) in TG neurons following DA or SKF incubation. Two adjacent traces are same at different time ranges. Ctrl and DA in B6: n = 83 and 18, respectively. Ctrl, DA and SKF in albino-B6: n = 43, 17 and 16, respectively. *P < 0.05, *vs* Ctrl, ^##^ *P* < 0.01, *vs* B6, in Chi-square test.

We focus on *Trpv1* and *Piezo2* as candidate genes in cutaneous dopaminergic modulations because these channels impact baseline withdrawal sensitivities to radiant heat and von Frey stimulation, respectively^29, 38^. *Trpv1* levels do not differ significantly in the TG from pigment B6 and albino-B6 (Fig. 4a); however, Ca^2+^-imaging demonstrates that a higher population of isolectin B_4_ (IB_4_)-positive neurons in pigment B6 mice respond to capsaicin (Fig. 4b). There was no difference in cell diameters of capsaicin-sensitive IB_4_-positive neurons between pigment B6 and albino-B6 mice (19.5 ± 1.3 and 18.9 ± 1.2 μm, respectively). Since IB_4_-positive sensory afferents predominantly innervate the epidermal layer^39^, high-sensitivity in the neuronal subpopulation likely contributes to the high heat sensation in skin. Following injection of dopamine or SKF38393 *in vivo*, *Trpv1* is up-regulated in both pigment B6 and albino-B6 (Fig. 4a). Dopamine incubation of IB_4_-positive neurons from pigment B6 mice does not change capsaicin sensitivity, as measured with Ca^2+^-imaging; however, both dopamine and SKF38393 increase capsaicin sensitivity in albino-B6 to a level similar to pigment B6 (Fig. 4b) without exhibiting a difference in the percentages of IB_4_-binding cells or Ca^2+^-influx amplitudes (Supplementary Fig. S3a,b). *Piezo2* expression is lower in pigment B6 than albino-B6 and is down-regulated following injection of dopamine or SKF38393 (Fig. 4c). Rapidly-adaptive (RA) mechanically-activated current in sensory neurons is generated by Piezo2 channel^38, 40, 41^. In patch-clamp recordings from cultured TG neurons, cell populations expressing the RA current are lower in pigment B6 than albino-B6 (Fig. 4d) without differences in RA current amplitudes (Supplementary Fig. S3c,d). RA cell population in albino-B6 is decreased by dopamine or SKF38393 incubation but not in pigment B6 (Fig. 4d).

## Discussion

**Figure 5:**
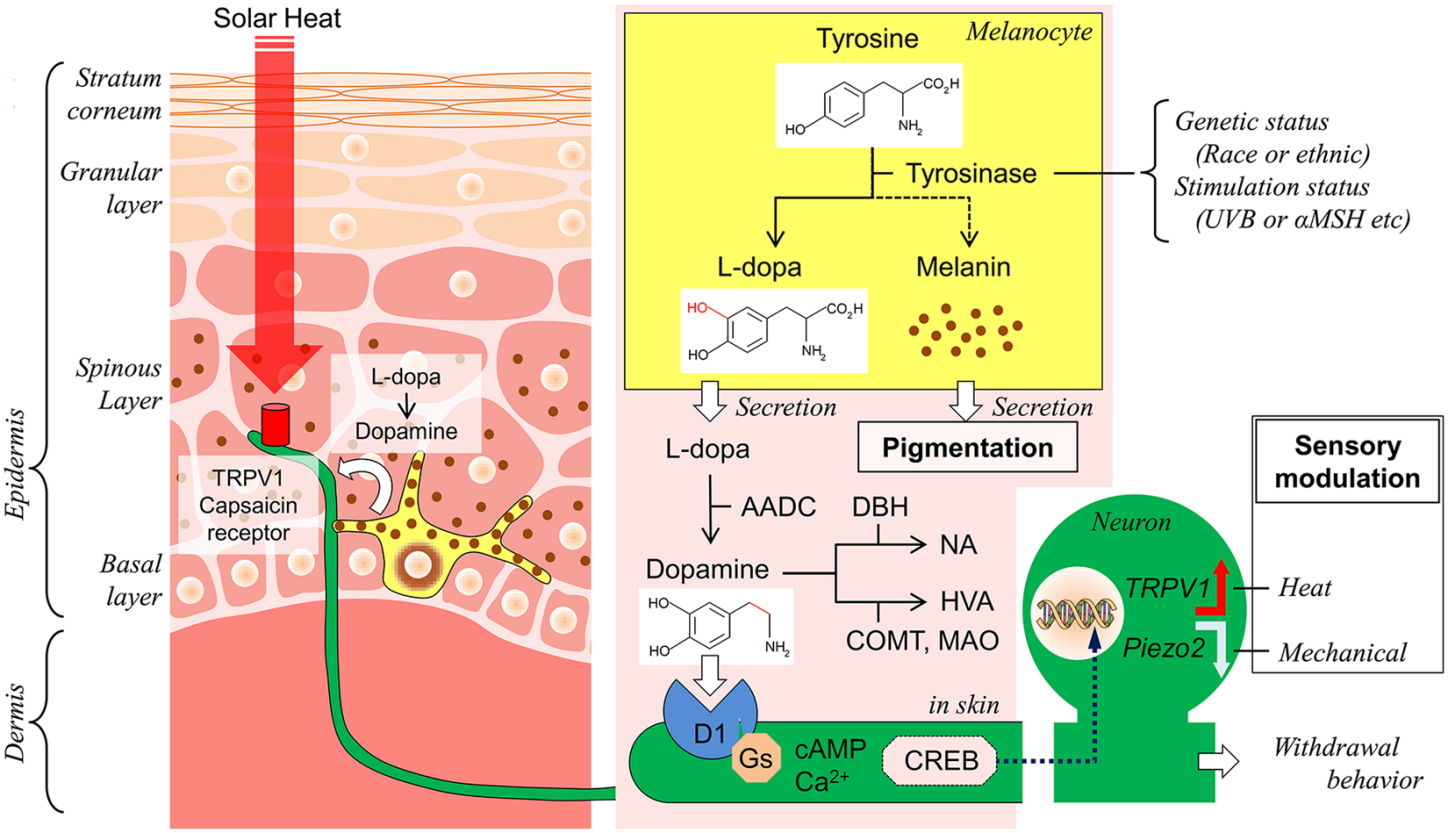
Mechanism of skin dopaminergic sensory modulation. The left schema represents dopaminergic signalling (white arrow) between nerve endings (green) and melanocytes (yellow) in structure of the epithelial skin. The right schema shows tyrosinase cascade in melanocytes, metabolism of L-dopa in extracellular space (pink) in the skin and presumed CREB-dependent gene modulation pathway from nerve endings to neuronal soma of primary sensory neurons, following dopaminergic D_1_ receptor activation. Tyrosinase activity is different among races or ethnicity (genetic status) and environment (stimulation status). A red arrow with TRPV1 and a white arrow with Piezo2 indicate up-regulation and down regulation, respectively. TRPV1 and Piezo2 are not always co-expressed although we draw both in the same neuronal soma. AADC, Aromatic L-amino acid decarboxylase; αMSH, α-Melanocyte stimulating hormone; COMT, catechol-O-methyltransferase; DBH, dopamine-β-hydroxylase; MAO, monoamine oxidase A; HVA, homovanillic acid; UVB, ultraviolet B.

We posit a peripheral dopaminergic mechanism to explain pigmentation-dependent differences in cutaneous sensitivity (see Fig. 5). During melanogenesis, melanocytes convert tyrosine to L-dopa by tyrosinase; L-dopa is converted to dopamine by DOPA decarboxylase. Melanocytes also produce melanin which is released into the surrounding tissue through dendritic processes and leads to pigmentation. Pigment B6 mice produce large amounts of L-dopa and dopamine in the skin; albino-B6 mice do not^23^. Dopamine stimulates D_1_-like receptors on peripheral nerves. Moreover, we propose that dopamine induces *Trpv1* upregulation and *Piezo2* downregulation in sensory neurons, resulting in higher heat sensitivity and lower mechanical sensitivity in pigment mice relative to albino mice. Similarly, lower heat sensitivity has been reported in less pigmented mice; these mice have dysfunction of MC1R that accelerates tyrosinase activity. Skin sensitivities in pigmented (brown) DBA/2 mice were intermediate between the black and albino strains, as shown in Fig. 2b and e. DBA/2 mice have a mutation in TYRP1 gene^42^. The TYRP1 mutation should not affect dopamine synthesis directly since TYRP1 is downstream of dopa decarboxylase^43^. The effect of the TYRP1 mutation on skin sensitivity might be due to its effect on melanocyte proliferation^21, 44^, which in turn affects the overall skin dopamine production. Consistent with these pigment-related results from animals, our meta-analysis demonstrated that the dark skin human cohort showed higher heat sensitivity in forearm skin relative to the light skin cohort.

We focused on dopamine signalling because, among the catecholamines, this molecule has the largest effect on heat and mechanical sensitivity. In the skin of newborn pigmented B6 mice, epidermal tyrosinase and melanin are no longer detectable 9 days after birth; skin and plasma dopamine are not detectable at 2-weeks. However, epidermal tyrosinase and melanin, along with L-dopa, remain detectable in the hair follicles at all ages^23^. We showed that antagonism of skin dopaminergic signalling using the D_1_-like receptor selective antagonist SCH23390 produced high heat and low mechanical sensitivity in pigment B6; the altered levels were similar to sensitivity levels in albino-B6. Together with the effects of the tyrosinase inhibitor kojic acid, these results suggest constitutive dopaminergic sensory modulation in cutaneous tissue of adult B6 mice. Because there are tight contacts between melanocytes and sensory afferents^22^ we propose that L-dopa is secreted by melanocytes into the epidermis and that dopamine activates mainly the neuronal D_1_-like receptor on sensory afferents, although dopamine cross activation on β-adrenoceptor is also possible^45^. Dopamine is then acutely degraded to homovanillic acid through the catecholamine degradation enzymes catechol-*O*-methyltransferase (COMT) and monoamine oxidase^31^ (Fig. 5). Norepinephrine produced through dopamine-β-hydroxylase^31^ is detected in the skin and plasma of mice, similar to L-dopa and dopamine, but concentration is dependent on tyrosine hydroxylase activity rather than tyrosinase activity^23^. Since β_2_-adrenocetor activation in melanocytes has been reported to promote melanogenesis^46^, secondary L-dopa production following epinephrine-promoted melanogenesis may indirectly modulate heat sensitivity on the day following injection of epinephrine.

D_1_-like receptors are coupled with G_s_ protein which activates cAMP^33^. Because the transcription factor cAMP response element binding protein (CREB) can be trafficked in a retrograde direction in peripheral sensory axons to cell somata^47^, CREB is a possible transcriptional candidate that mediates skin dopaminergic gene modulations. CREB binding sites are not detected within the two promoter regions, P1 and P2, of the rat TRPV1 genes^48^. Similarly, putative CREB sites are not detected within the P1 and P2 regions (−1007–−1 bp upstream of the transcription initiation site) of the upstream region of mouse TRPV1 gene based on an online JASPAR analysis; however, the putative site is detected in the more upstream position (−1708 bp). Two putative CREB sites (−1751 and −340 bp) are detected in the upstream region of human TRPV1 gene. The position of the human putative CREB site (−1751 bp) is similar to that in mouse (−1708 bp). Therefore, dopaminergic stimulation may directly modulate *Trpv1* expression via CREB (Fig. 5).

Previous evidence suggests that chronic D_2_-like receptor stimulation in human and mice haplotypes with lower activity of COMT elicits higher pain sensitivity due to central plasticity including reduced capacity to activate the pain-inhibitory µ-opioid receptor in various brain regions^25, 49–52^. However, our proposed skin dopaminergic sensory mechanism provides a new explanation for higher heat sensitivity in the COMT haplotypes; higher dopamine levels in skin under lower COMT activity lead to greater heat sensitivity via D_1_-like receptor activation in the peripheral nervous system. A study in healthy volunteers shows that the centrally-acting D_2_ receptor antagonist does not change heat sensitivity in the palm^53^. We infer from these findings that dopaminergic modulation of peripheral sensitivity is not centrally-mediated. Therefore, the skin dopaminergic sensory mechanism may explain the previous observation that COMT haplotypes coding for low COMT activity increased thermal pain sensitivities^51, 52^. The dopaminergic mechanism may also contribute to heat hypersensitivity in sunburn after UVB overexposure^54^. Reported attenuation of mechanical allodynia following melanoma cell inoculation^55, 56^ may be due to the L-dopa released from the melanoma. Disturbance of the dopaminergic signalling in the peripheral may also contribute to the upregulation of Piezo2 and mechanical allodynia after nerve injury^41^.

## Methods

### Meta-analysis

A systematic search of published studies in the Pubmed database from January, 2000 through May, 2014 was performed using the following key words and syntax; #1: [Date-Publication] 2000/01/01–2014/12/31, #2: pain, #3: race OR racial OR ethnic and #4: mechanical OR tactile OR thermal OR heat. Sufficient data from 11 studies were available to conduct separate meta-analyses for heat pain intensity (% of visual analogue scale) to heat stimulation of 48 or 49 °C (if both temperatures were tested, data from 48 °C stimulation was analyzed), heat pain threshold (the first painful temperature; °C) and heat pain tolerance (intolerable temperature; °C) to a heating thermode at 48 or 49 °C on the forearm skin and pressure pain threshold (the first painful pressure; kPa) in the trapezius and masseter muscles.

### Publicly available database for nocifensive behaviours

Data from the “Heritability of Nociception Project”, which is publicly available on the Jackson Laboratory’s Mouse Phenome Database Website (http://www.jax.org/phenome; Projects: Mogil1 and Mogil2), were used as the source data for comparison of nocifensive behaviours in inbred mouse strains. The database included mechanical nociceptive assays such as tail clip tests and von Frey tests, thermal sensitivity assays such as hot-plate tests and Hargreaves’ tests, and licking after chemical applications in 12 inbred mouse strains (please see the “Heritability of Nociception Project” for experimental details). Data for the C57BL/10 strain and the dynorphin-induced mechanical withdrawal assay were excluded from the analysis because complete data sets were not available. Based on body colour, 11 strains were selected and divided into pigmented (C3H/He, C57BL/6, C58, CBA, DBA/2 and SM) and unpigmented (129P3, A, AKR, BALB/c and RIIIS) groups. All strains were assigned a *z*-score for each nociceptive assay. Detailed experimental methods, protocols, and comparative results can be found in previous publications^28, 57, 58^. Mean values for each strain were downloaded from the website and the data were transformed into a *z*-score using the formula *z* = (χ − μ)/σ, where χ is the strain mean score of the assay, μ is the mean score of all 11 strains in the assay and σ is the standard deviation across all strains in the assay^25^. For all calculated *z*-scores, the greater values reflect greater nociceptive sensitivity. For heat nociceptive sensitivity in the tail or hind paw, the sum *z*-scores were obtained by adding results from 2 heat nociceptive assays (tail withdrawal latency to 47 °C and 49 °C water immersion test; paw withdrawal latency to 59 °C in hot-plate test and Hargreaves’ test) were used. For mechanical nociceptive sensitivity, *z*-scores of tail clip tests and von Frey tests at the hind paw were used. Furthermore, according to the reported classification of pain assays^28^, all nociceptive assays were classified into 5 modalities (thermal, chemical, afferent-dependent, thermal hypersensitivity and mechanical hypersensitivity). The former two modalities are designated as physiological baseline pain sensitivities in naïve mice and the latter three modalities are designated as pathological pain sensitivities in inflammatory and neuropathic pain models of mice. In the former 4 modalities, the sum *z*-score in each modality was obtained by summing 2 to 6 nociceptive assays. For capsaicin- and formalin-induced pain, the sum *z*-scores were obtained by adding the results from the two different capsaicin and formalin tests.

### High-performance liquid chromatography (HPLC)

The melanoma cell line WM164 was cultured in Dulbecco’s Modified Eagle Medium (DMEM) with 10% fetal bovine serum at 37 °C with 5% CO_2_. After the cells were grown into confluence, the cell culture media was replaced with 3 ml DMEM containing either 0.01% dimethyl sulfoxide (DMSO) (as control) or 30 μM α-melanocyte-stimulating hormone (Abcam, MA, USA) with 0.01% DMSO. α-MSH is well known to activate tyrosinase activity in melanoma cells^59^. Supernatant was collected 72 hours after drug application. Samples were centrifuged and stored in −20 °C before analysis. HPLC coupled to ultraviolet detection was employed for catecholamine measurement. Samples (10 μl) were injected into the HPLC system with a Waters 2795 Separations Module microsampler (Waters Corporation, MA, USA). Mobile phase was pumped at 1 ml/min and consisted of 2% acetonitrile in 0.1 M KH_2_PO_4_ at pH 4.5. Samples were passed through a 100 × 4.1 mm C18 column (Waters Corporation). A Waters 2487 Absorbance Detector was set at 210 nm (Waters Corporation). For these experiments, L-dopa was purchased from Abcam and dopamine, norepinephrine and epinephrine were purchased from Sigma-Aldrich (CA, USA). Catecholamine standards were freshly prepared in DMEM and 0.01% DMSO. The L-dopa standard calibration concentration ranged from 0 to 500 μM. A linear relationship was observed between L-dopa concentrations and the area under the curve (*R* ^2^ = 0.991) or peak height (*R* ^2^ = 0.985). Identification of each catecholamine in the samples was confirmed by spiking the sample with the standard. Area under the curve was used to quantify the concentration of L-dopa released in the different experimental treatments.

### Animals

We utilized female B6 and B6 (Cg)-*Tyr* ^*c*−*2J*^ mice (6–10 weeks old, Charles River Laboratories, NY, USA) weighing 16–20 g., exposed to a 12 hour light-dark cycle, and kept in a temperature-controlled room with food and water ad libitum. All procedures involving animals were approved by the New York University Institutional Animal Care and Use Committee (IACUC) under protocol # 160908–01, in accordance with the Guide for the Care and Use of Laboratory Animals published by the U.S. Institute for Laboratory Animal Research (8^th^ edition).

### Drug injections

Under light general anaesthesia with isoflurane (Summit Medical Equipment Company), drug-containing phosphate-buffered saline (PBS) at 50 μl was subcutaneously injected over a 5 sec period, into the tested regions of the whisker pad and hind paw. The needle was inserted 7 mm subcutaneously. A 30-G needle was used to reduce the nocifensive effect of the needle-prick. Repetitive drug injection was performed 3 times, once per day at 1 day intervals.

L-dopa, dopamine, norepinephrine and epinephrine were dissolved in PBS. The D_1/5_ selective agonist SKF38393 (Spectrum Chemical Mfg Corp, CA, USA), the D_2/3_ selective agonist quinpirole (Tocris, Bristol, UK), the D_4_ selective agonist PD168077 (Tocris), the D_1_/D_5_ selective antagonist SCH23390 (Tocris), the COMT inhibitor entacapone (Abcam)^60^ and the representative tyrosinase inhibitor kojic acid (MP Biomedicals, OH, USA)^61^ were dissolved in DMSO as stock solutions and then diluted to 100 or 50 times in PBS just before injections. PBS with 1% or 2% or no DMSO was used as controls for these drug injections. The concentrations of L-dopa and catecholamines were 50 nmol 50 μl^−1^, based on a previous study showing moderate heat hypersensitivity in the hind paw following local injection of norepinephrine at 20–200 nmol 30 μl^−1^ ^62^. The three dopamine receptor agonists were administered at a lower concentration (15 nmol) than that of dopamine, based on a previous *in vitro* study showing that 30% lower concentrations of the agonists induced similar neuronal activity to dopamine^63^. The SCH23390 and entacapone concentrations were 50 nmol and the kojic acid concentration was 1.5 μmol.

### Mouse behaviours

In all behavioural experiments, animals were assigned to groups with a randomized block design, and measurements were performed with the experimenter (K.O.) blind to the drug treatments. To apply heat and mechanical stimulation to the whisker pad and measure withdrawal, mice were restrained in a plastic tube, according to a previous study^29^. The retention tube was made from a 50-ml conical tube by cutting a 13 mm diameter hole in the bottom of the tube, and then cutting the tube off at the 30-ml graduation line. Mice were habituated to enter and stay in the retention tube for three sessions (once per day) prior to nociceptive testing. For the hind paw tests, mice were placed in plastic chambers on a glass surface (25 °C, for heat stimulation) or a raised metal mesh platform (for mechanical stimulation) and acclimated for 10–30 min prior to measurements.

For thermal sensitivity, head or paw withdrawal latency was measured using a thermal stimulator (Hargreaves’ Apparatus; Department of Anaesthesiology, University of California, San Diego), according to the general method of Hargreaves’ test for the hind paw^64^ and a modified method for the whisker pad^29^. To measure heat sensitivity in the whisker pad, a thick-paper cone with a 7 mm diameter opening was attached to the thermal stimulator. The thick-paper cone was used to maintain a constant distance (8 cm) between the heat-filament and the whisker pad. The radiant heat source was focused on the hind paw or whisker pad. For mechanical sensitivity, head or paw withdrawal threshold was measured by using a logarithmically increasing set of von Frey filaments (Semmes Weinstein monofilament set; Briggate Medical, Braeside, Australia), ranging in gram force from 0.07 to 26 g. The filaments were applied to the hind paw or whisker pad with sufficient force to cause a slight bending of the filament one per 1 second, at least, 5 times. If mechanical stimulation showed a positive response characterized as a rapid withdrawal, the same stimulation was confirmed twice after checking for no response with the lower force stimulation. The heat and mechanical sensitivities were measured as the average of 3–4 trials per animal taken >5minutes apart. A cutoff was set at 20 seconds for heat stimulation or 26 g for mechanical stimulation to avoid tissue damage.

The heat stimulation was applied in the first hour and the mechanical stimulation was applied in the second hour. When testing for acute drug effects, the tests were performed 30–40 min after isoflurane anaesthesia. The acute effects of the agent on cutaneous sensitivity were represented as the percent change relative to the initial values before drug injection. When testing for late drug effects, the tests were performed on day 1, day 4 and day 14 following the last injection.

### RNA quantification

The trigeminal ganglia (TG) were removed under isoflurane anaesthesia. Ten mg of fresh frozen TG (5 samples from 5 animals in each mouse strain) was homogenized with a Mini Beadbeater-1 (BioSpec Products, OK, USA) and subjected to RNA/DNA extraction with AllPrep DNA/RNA Kit (Qiagen, CA, USA). mRNA was reverse transcribed with Random Hexamers (Applied Biosystems, CA, USA). Five replicates were performed in each strain. A 2 µl cDNA aliquot was amplified on the Mx3005 P qPCR system (Agilent Technologies, CA, USA) according to manufacturer’s recommendations with the Taqman gene expression assay for *Trpv1* (TRPV1 gene; Mm01246302_m1) and *Piezo2* (*Fam38b*; Piezo2 gene; Mm01265861_m1), which did not detect residual genomic DNA (Applied Biosystems). Mouse *ACTB* (Applied Biosystems) was used as the endogenous control. The delta-delta CT method was used to quantify relative expression. The assays were carried out in duplicate and the respective relative amounts of *Trpv1* and *Piezo2* to *ACTB* were calculated in each sample.

### Immunofluorescence

For immunohistochemistry, the TG were dissected from mice after perfusion with 4% paraformaldehyde in phosphate-buffered saline (PBS) under deep isoflurane anaesthesia and cut in the horizontal plane at 8 μm thickness in a cryostat. After sectioning, TG sections were rinsed with PBS and blocked with SuperBlock solution (Thermoscientific, PA, USA) for 1 hour, and then incubated overnight in the primary antibodies: D_1_ (anti-rabbit polyclonal, 1:600; ab85608, Abcam) and D_5_ (anti-rabbit polyclonal, 1:500; 324408, Calbiochem, MA, USA). Three mice from B6 or albino-B6 were used in each experimental group. After incubation with the primary antibody, sections were rinsed in PBS 5 times for 5 minutes each and then incubated in chicken anti-rabbit Fluor® 488 (1:1000; Invitrogen, CA, USA) for 2 hours at room temperature. Thereafter, sections were washed with PBS and coverslipped with UltraCruz mounting medium (Santa Cruz Biotechnologies, TX, USA). Control tissue sections were incubated with secondary antibody only or pre-absorption of the primary antibody; control sections showed no positivity (data not shown). Images were taken under an epifluorescence microscope (Eclipse Ti; Nikon, Tokyo, Japan). Three random sections from each slide were used for quantification of each staining. Data analysis was performed using Nikon Element software, which allowed both single and merged picture acquisition.

### TG cultures

TG neurons were dissociated according to an established protocol^65^. After deep anesthetization with isoflurane, the TG were removed and transferred into a culture medium (Ca^2+^ and Mg^2+^-free Hanks balanced salt solution, Invitrogen). After mincing the tissue into 10–12 pieces, the tissues were incubated in collagenase type 2 (3.3 mg ml^−1^; Worthington Biochemical, NJ, USA) and dispase II (4.7 mg ml^−1^; Roche) for 20 minutes and then in papain (20 U ml^−1^, Worthington Biochemical) for 20 minutes at 37 °C. After trituration and centrifugation, the cell pellet was resuspended with F-12 (Invitrogen) containing 5% fetal calf serum and plated on laminin-coated coverslips. The cells were incubated at 37 °C in a humidified 5% CO_2_ chamber for 1 day (20–28 hours). For Ca^2+^-imaging and whole-cell patch-clamp recordings, dissociated neurons were preincubated with dopamine at 100 μM or dopamine receptor agonists at 30 μM for 1 day before recordings.

### Ca^2+^-imaging

Protocol for Ca^2+^ imaging was previously described^66, 67^. Cultured TG neurons were incubated in a clear culture medium (Ca^2+^ and Mg^2+^-containing DMEM) with 10 µM fura-2AM (Life Technologies, OR, USA) for 30 minutes before Ca^2+^ imaging. Cover slips were mounted on a custom aluminium perfusion block and viewed through an inverted fluorescence microscope (Nikon Eclipse TS100, NY, USA). Fluorescence was excited by UV light at 340 and 380 nm alternately, and the emitted light was collected by using a CoolSnap camera attached to a Lambda LS lamp and a Lambda optical filter changer (Sutter Instrument Company, CA, USA). Ratiometric measurements were made using the computer software Simple PCI (Compix, PA, USA) every 1 second. Solutions were delivered by a solenoid-controlled 4-channel perfusion system at a flow rate at 1 mL min^−1^. Capsaicin (Sigma-Aldrich) at 1 μM, dopamine at 100 μM and dopamine receptor agonists at 30 μM were applied for 2 minutes and KCl solution (added 50 mM KCl) was applied at end of the experiment to confirm that the recorded cells were active neurons. Stock solutions of drugs were dissolved in DMSO. The stock solution was diluted to 0.1% with DMEM (as perfusion solution). DMSO alone at 0.1% was confirmed to have no effect on intracellular Ca^2+^ concentration in TG neurons. Cells were judged to be responsive if the ratio value increased by greater than 0.1 (Δ ratio unit of F340/F380) after chemical application.

### Whole-cell patch-clamp recording

Whole-cell patch-clamp recording procedures were described in previous studies^29, 66^. Dissociated neurons were cultured for one day and then coverslips with neurons were transferred to a recording chamber superfused continuously with external solution containing the following (in mM): 140 NaCl, 4 KCl, 2 MgCl_2_, 2 CaCl_2_, 10 glucose and 10 Hepes (pH 7.3 with NaOH, 320 mOsm kg^−1^ with sucrose), at room temperature. Cell sizes of recorded neurons ranged in diameter from 11–34 μm. Patch pipettes were double-pulled (P-2000, Sutter, CA, USA) from quartz glass capillaries (Q100–50–10, Sutter). Pipettes were adjusted to 3–7 MΩ when filled with a pipette solution (in mM): 145 KCl, 3 MgCl_2_, 2.25 CaCl_2_, 1 EGTA, 10 Hepes (pH 7.3 with KOH, 310 mOsm kg^−1^ with sucrose). After establishing the whole-cell configuration, the voltage was clamped at −60 mV using Axopatch 200B amplifier (Molecular Devices, CA, USA) and controlled by Clampex software (pClamp 10.2; Molecular Devices). Drugs were applied using a fast-step SF-77B superfusion system (Warner Instrument, CT, USA) with a three-barrelled pipette placed near the cell.

Mechanical stimulation was performed by a piezo-controlled micromanipulator (Nanomotor MM3A, Kleindiek Nanotechnik, Reutlingen, Germany). Mechanical stimuli to neurons were applied using a heat-polished glass pipette (tip diameter of 2–5 μm; estimated velocity of 10 μm/ms) as a stimulation probe, which was positioned at an angle of 45 degrees to the surface of the dish. The amount of probe displacement was controlled by using macro-based software commands (NanoControl; 1 step = 1 μm) and gradually increased by one increment at intervals of 60 seconds. The stimulus was applied for 250 ms. We analyzed data from steps 1–5 (*i.e*., 1–5 μm displacements) because use of more than 6 steps frequently induced G-seal rupture or incomplete recovery to base current level (>20% in membrane resistance). If the recorded cells showed mechanically-activated current of >30 pA, the cells were deemed to be sensitive to the mechanical stimulation. The falling phase of mechanically-activated currents were fitted with single exponential functions to measure their inactivation time constants (*τ*) and classified into three types of current (rapidly-adapting (RA: *τ* < 10 ms), slowly-adapting (SA: *τ* > 10 ms) and mixed current), according to a previous study^41^.

### Sampling and statistical analyses

In meta-analysis for collected human studies, all data suitable for pooling was analyzed by RevMan 5.4 software (Cochrane Collaboration) using random-effects analysis. For each study, the standardized mean difference and 95% confidence interval were calculated. Heterogeneity among studies was tested by using the Cochran *Q* statistic (Chi-square value) and was quantified by using the *I* ^2^ statistic. The suggested thresholds for low, moderate and high degrees of *I* ^2^ heterogeneity are 25%, 50% and 75%, respectively^68^. For *in vivo* studies, including analyses of the publicly available database, sample sizes were more than 4 per group, which is commonly required to obtain statistical significance, while *in vitro* studies utilized a minimum of three experimental replicates. For correlation analyses across mouse strains with two nocifensive behaviours, two-sided Pearson correlation test was used. Data are presented as the mean ± s.e.m.; n represents the number of mice or neurons tested. No animals/samples were excluded from analysis. Behavioural data showing in Fig. 3 and supplemental Fig. S1 used the same baseline. Data for the prolonged effect of drug injections (days following injection) were presented as actual measurement values such as withdrawal latency and withdrawal thresholds (Fig. 3); the acute effect of drugs (hours following injection) was presented as a percentage change from baseline values for better visualization (Fig. S1). All statistical tests were performed using GraphPad Prism version 5 for Windows (GraphPad Software, La Jolla, CA) and all reported statistical analyses were justified based on sample size, homogeneity of variance and normal distribution of the data. For statistical analyses of the different animal groups, two-sided Student’s unpaired *t*-test was used. For statistical analyses of the time-course of drug effects for the behavioural experiments, two-way repeated measure ANOVA test was used; Sidak’s multiple comparison test was used as a *post hoc* test for each day. For statistical analysis of an acute drug effect, Dunnett’s multiple *post hoc* test, following one-way ANOVA test, was used. Fisher’s exact test and Chi-square test were used to compare percentages of responders between two groups and among more than two groups, respectively. Significance was presumed at *P* < 0.05.

All original data generated and analyzed during this study are included in this published article and its Supplementary Information files.

## Electronic supplementary material

Supplementary Information
