# Supplementary material for: Cutaneous pigmentation modulates skin sensitivity via tyrosinase-dependent dopaminergic signalling
Source: Sci Rep. 2017 Aug 23;7:9181. doi: 10.1038/s41598-017-09682-4 (PMC5569050; doi:10.1038/s41598-017-09682-4)
Supplement: Supplementary file 1 — Supplementary Information [file 41598_2017_9682_MOESM1_ESM.pdf]

Supplementary figures

Cutaneous pigmentation modulates skin sensitivity via tyrosinase-dependent dopaminergic signalling

Kentaro Ono, Chi T Viet, Yi Ye, Dongmin Dang, Suzuro Hitomi, Takashi Toyono, Kiyotoshi Inenaga, John C. Dolan & Brian L. Schmidt

Table S1 | Characteristics of the studies included in the meta-analysis

Study name	Published year	Age range (mean)	Sex	Race/ethnicity	Sample size (dark/light)	Pain measurements
Sheffield (*22*)	2000	20-73 (38.0)	F/M mixture	AA, W	51 (24/27)	HPI
Yosipovitch (*23*)	2004	19-59 (35.6)	F/M mixture	Chi, I, M	49 (29/20)	HPTh
Campbell (*24*)	2005	NR (21.1)	F/M mixture	AA, W	120 (62/58)	HPI, HPTh, HPTo
Watson (*25*)	2005	Adults (NR)	M only	SA, WB	40 (20/20)	HPI , HPTh
Mechlin (*26*)	2007	18-47 (NR)	F and M	AA, NHW	84 (45/39)	HPTh, HPTo
Rahim-Williams (*27*)	2007	18-53 (24.0)	F and M	AA, H, NHW	206 (124/82)	HPTh, HPTo, PPTh
Grewen (*28*)	2008	NR (27.4)	F only	AA, NHW	48 (25/23)	HPTh, HPTo
Wang (*29*)	2010	19-47 (NR)	F/M mixture	AA, H, C, EA	40 (20/20)	HPTh
Sibille (*30*)	2011	18-45 (23.1)	F/M mixture	AA, NHW	142 (23/119)	HPI, HPTh, HPTo, PPTh
Hastie (*31*)	2012	18-54 (23.7)	F/M mixture	AA, H, NHW	247 (160/87)	HPI, HPTo, HPTh, PPTh
Riley III (*32*)	2014	45-76 (57.6)	F/M mixture	NHW, NHB	191 (53/138)	HPI, HPTo, HPTh, PPTh

Mean age (in years): Age range: NR, not reported. Sex: F, females; M, males. Race/ethnicity: AA, African Americans; C, Caucasians; Chi, Chinese; EA, East Asians; H, Hispanics; I, Indians; M, Malays; NHW, non-Hispanic Whites; SA, South Asians; W, Whites; WB, White British. Sample size: Dark, dark skin group including AA, H, I, M and SA; Light, light skin group including C, Chi, EA, NHW, W and WB, based on native skin color distributions (*21*). Pain measurement: HPI, heat pain intensity at 48 or 49°C; HPTh, heat pain threshold; HPTo, heat pain tolerance; PPTh, pressure pain threshold in trapezius and/or masseter.

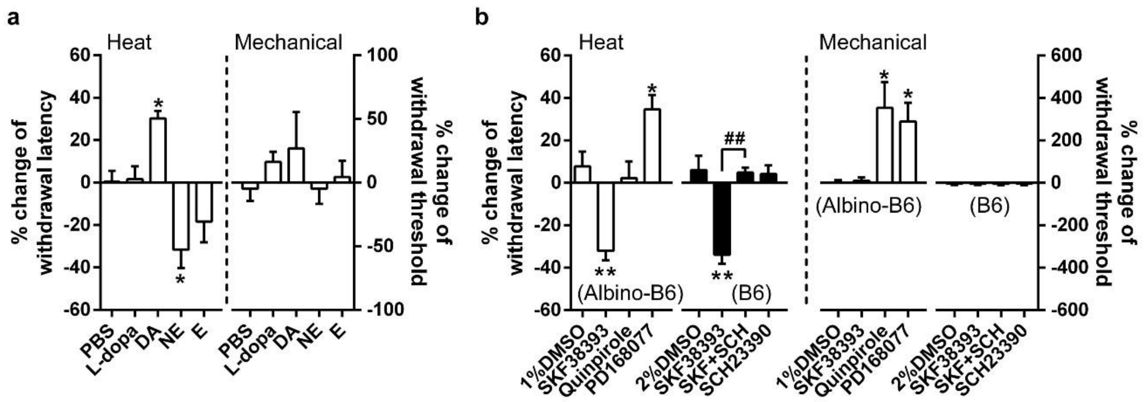

Figure S1 | Acute effects of L-dopa, catecholamines, dopamine receptor agonists and antagonists on cutaneous sensitivities in mice. (**a**) Percent changes of withdrawal latency to heat stimulation (heat sensitivity) 1 hour following injections of PBS, L-dopa, DA, NE and E and threshold to von Frey stimulation (mechanical sensitivity) in 2-3 hours after injections of the same agents in albino-B6. **P*<0.05, *vs* PBS, in Dunnett's multiple *post hoc* test, following one-way ANOVA test. (**b**) Percent changes of withdrawal latency to heat stimulation and mechanical withdrawal threshold from 30-40 min after injections of dopamine receptor agonists and antagonists in B6 and albino-B6. * *P*<0.05, ***P*<0.01, *vs* controls (1% or 2% DMSO in PBS), in Dunnett's multiple *post hoc* test, following one-way ANOVA test. ##*P*<0.01, *vs* a SKF38393-injected group, in *t*-test. Each experimental group, n=5. All bars show mean ± s.e.m.. The analyses present % change against pre-values (before drug injections), which are presented in Fig. 3.

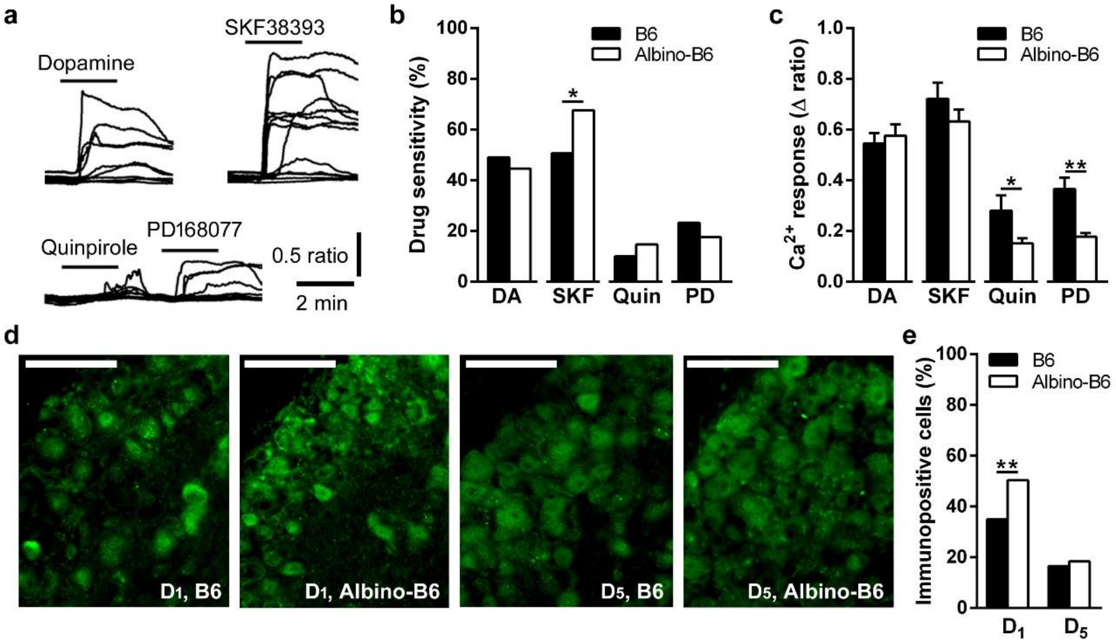

Figure S2 | Dopamine receptor expression in cultured TG neurons of B6 and albino-B6. (**a**) Ca^{2+} responses by dopamine and receptor-specific agonists; DA concentration was 10 μM. The $D_{1/5}$ agonist SKF38393 (SKF), the $D_{2/3}$ agonist quinpirole (Quin) and the D_4 agonist PD168077 (PD) at 30 μM. Quin and PD were applied to the same neurons. (**b**) Sensitivities to dopaminergic agents. DA, SKF, Quin and PD in B6: total n=75/153, 45/89, 9/90 and 21/90, respectively. DA, SKF, Quin and PD in albino-B6: total n=70/157, 69/102, 15/102 and 18/102, respectively. *P<0.05, in Fisher's test. (**c**) Peak of dopaminergic agent-induced Ca^{2+} responses. *P<0.05, **P<0.01, in t-test. All bars show mean ± s.e.m.. (**d**) Immunoreactivities to D_1 and D_5 dopamine receptor in the mouse TG neurons. Scale bar, 100 μm. (**e**) Cell populations showing D_1 and D_5 immunoreactivities in total neurons of B6 (n=86/246 and 55/171, respectively) and albino-B6 (n=148/294 and 73/233, respectively). **P<0.01, in Fisher's test.

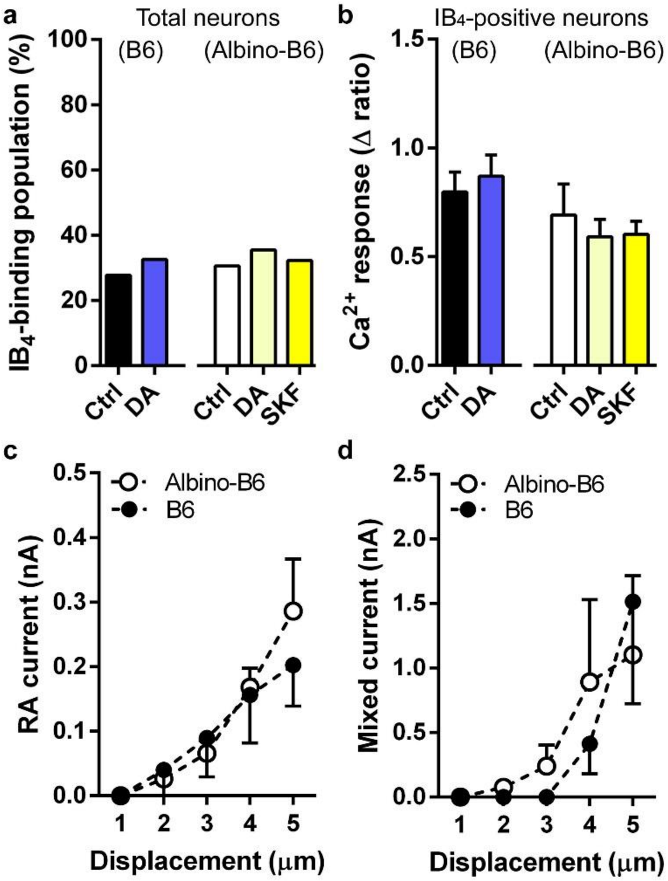

Figure S3 | Effects of dopaminergic stimulations on capsaicin-induced Ca^{2+} responses and mechanically-activated currents in cultured TG neurons. (**a**) IB_4 binding cell populations. Ctrl and DA in B6: total n=35/126 and 45/138, respectively. Ctrl, DA and SKF in albino-B6: total n=30/98, 55/155 and 40/124, respectively. (**b**) Peak of capsaicin-induced Ca^{2+} responses in IB_4-positive neurons. Ctrl and DA in B6: n=27 and 29, respectively. Ctrl, DA and SKF in albino-B6: total n=12, 37 and 23, respectively. (**c**) Displacement-dependency of rapidly-adapted (RA) currents. (**d**) Displacement-dependency of mixed currents with RA and slowly-adapted currents. All bars and plots show mean ± s.e.m..
